# The Effect of FOXC2-AS1 on White Adipocyte Browning and the Possible Regulatory Mechanism

**DOI:** 10.3389/fendo.2020.565483

**Published:** 2020-10-29

**Authors:** Yan Wang, Siyu Hua, Xianwei Cui, Yan Cao, Juan Wen, Xia Chi, Chenbo Ji, LingXia Pang, Lianghui You

**Affiliations:** ^1^ Nanjing Maternity and Child Health Care Institute, Women’s Hospital of Nanjing Medical University, Nanjing Maternity and Child Health Care Hospital, Nanjing, China; ^2^ Department of Child Health Care, Women’s Hospital of Nanjing Medical University, Nanjing Maternity and Child Health Care Hospital, Nanjing, China

**Keywords:** lncRNA, FOXC2-AS1, human white adipocytes, Browning effect, autophagy

## Abstract

Obesity has become a worldwide epidemic, and obesity-related problems are becoming more severe in public health. Increasing brown adipose tissue (BAT) mass or/and activity in mice and humans has been demonstrated to help lose weight and improve whole-body metabolism. Studies on the conversion of white adipose tissue (WAT) to BAT under certain conditions have provided new possibilities for treating obesity and the related disorders. It has been established that long non-coding RNAs (lncRNAs) play an important role in the regulation of mouse adipocyte differentiation and thermogenic programs; however, the function and potential mechanism of lncRNA in the process of human white adipocyte browning remains unclear. In the present study, we identified a lncRNA called Forkhead Box C2 antisense RNA 1 (FOXC2-AS1), which was first identified in osteosarcoma, and it was highly expressed in human adipocytes but decreased during the white adipocyte differentiation program. FOXC2-AS1 expression was also induced by the thermogenic agent forskolin. Lentivirus-mediated overexpression of FOXC2-AS1 in human white adipocytes did not affect lipid drop accumulation, but significantly promoted the browning phenotype, as revealed by the increased respiratory capacity and the enhanced protein expression levels of brown adipocyte-specific markers. In contrast, inhibiting FOXC2-AS1 with small interfering RNA led to attenuated thermogenic capacity in human white adipocytes. RNA-sequencing analysis and western blot were used to identify a possible regulatory role of the autophagy signaling pathway in FOXC2-AS1 to mediate white-to-brown adipocyte conversion. The autophagy inhibitor 3-methyladenine restored the reduced UCP1 protein level and thermogenic capacity caused by inhibiting FOXC2-AS1. Overall, the present study characterized the potential role of FOXC2-AS1 and further identified a lncRNA-mediated mechanism for inducing browning of human white adipocytes and maintaining thermogenesis, further providing a potential strategy for treating obesity and related disorder.

## Introduction

Obesity and its related complications such as type 2 diabetes mellitus, coronary heart disease and obstructive sleep apnea, have been considered significant health problems (1). Although dietary management (2), exercise (3), and pharmacological intervention (4) have been proven to control weight, these approaches are largely inefficient for maintaining healthy long-term weight loss. Therefore, effective therapies for treating obesity and related metabolic disorders are needed.

The two main types of adipose tissue are white adipose tissue (WAT) and brown adipose tissue (BAT) (5). WAT principally stores excess energy as lipids, whereas BAT is a specialized adipose tissue that converts nutrients, such as glucose and fatty acids, into heat (non-shivering thermogenesis) depending on activating uncoupling protein 1 (UCP1) in mitochondrial membranes (6) or not (7). Increased BAT mass (8) or activity (9, 10) has been demonstrated to have the anti-obesity and anti-diabetic effects in mice, as revealed by decreased body weight, improved insulin resistance, enhanced glucose metabolism, and augmentation of whole-body energy expenditure. Similarly, BAT has the capability to elevate energy expenditure, reduce lipid accumulation, and enhance glucose homeostasis in adult humans (11, 12). However, the therapeutic prospects of BAT are limited because its mass and activity reach a peak during the neonatal stage and then decrease in adults, particularly in humans. Physiological stimulation, such as cold exposure (13), pharmacological intervention [rosiglitazone (14) and a β-adrenergic agonist (15)], and natural compounds [berberine (16), flavonoids (17), and resveratrol (18)] have been demonstrated to induce brown-like adipocytes from WAT (browning) in mice. These observations convinced us to identify the possible regulators or underlying mechanisms involved in the browning process that may have potential as anti-obesity treatments.

Long non-coding RNAs (lncRNAs) are RNA molecules whose transcripts are longer than 200 nt in length and they do not encode a protein (19). Mounting evidence indicates that lncRNAs exerted essential roles in diverse biological processes, including X chromatin activation (20), embryonic development (21), cell differentiation (22), and the pathogenesis of multiple tumors (23). Comprehensive lncRNA catalogs have been enriched in various types of adipose tissues using integrated RNA-sequencing technology (24). Similarly, many studies have fully established the potential role and identified the importance of lncRNAs in the adipogenic differentiation of adipose-derived stem cells (25), preadipocytes (26), and cell lines (27) in mouse or human cell models. However, the exact role or possible regulatory mechanisms of lncRNAs involved in the conversion of white adipocytes to brown adipocytes have not been revealed. Although mouse genome-derived lncRNAs, such as Brown fat lncRNA 1 (Blnc1) (24), brown adipose tissue enriched long non-coding RNA 10 (BATE10) (28), AK079912 (29), and Gm13133 by our previous study (30), have helped researchers understand the browning of white adipocytes, the annotation and functional exploration of lncRNAs in human adipocytes are more meaningful to uncover the relevance to human biology.

In this study, we determined the role and possible mechanism of FOXC2-AS1 during white adipocyte differentiation and browning. The results showed that overexpressing FOXC2-AS1 did not affect adipogenesis of human preadipocytes but promoted the browning phenotype, whereas knockdown of FOXC2-AS1 inhibited the process.FOXC2-AS1 may participate in the autophagy signaling pathway to mediate white-to-brown adipocyte conversion. These observations further deepen our understanding of the role and mechanism of lncRNAs during the browning process of white adipocytes and provide a potential molecular target to the treat of obesity and related metabolic disorders.

## Material and Methods

### Human Subjects

Subcutaneous WAT samples were obtained from the patients undergoing abdominal liposuction at Nanjing Maternal and Child Health Hospital. Signed informed consent for the tissue was obtained. These patients did not have any malignancies, endocrine diseases or severe systemic illness. The details of the participants such as gender, age, body mass index, and health status are listed in **Supplemental Table S1** as described previously (31). This study was approved by the Human Research Ethics Committee of Nanjing Maternity and Child Health Care Hospital (permit number [2020] KY-027).

### Isolation of Preadipocytes Derived From Subcutaneous White Adipose Tissues

Primary human preadipocytes were isolated from adult subcutaneous fat as described previously (32). The segregated tissues were minced and digested in DMEM (Gibco, Grand Island, NY, USA) with 2% collagenase I (Sigma-Aldrich, St. Louis, MO, USA) for 1 h and the digestive suspension was shaken every 5 min. After digestion, the cell suspension was filtered through a 70 μm cell strainer (Thermo Fisher Scientific, Waltham, MA, USA), and then the filtered solution was centrifuged at 2000 rpm and 25 °C for 10 min. The tubes were rinsed with PBS (Gibco) twice, and the cell pellets were resuspended in DMEM complete medium supplemented with 10% fetal bovine serum (Gibco) and 1% penicillin/streptomycin solution (Gibco), seeded on a 6-well plate, and then cultured at 37 °C and 5% CO2.

### Cell Culture and Cell Differentiation

Human preadipocytes from subcutaneous adipose tissue before passage 5 were thawed and maintained in growth medium (PAM; ScienCell Research Laboratories, Carlsbad, CA, USA) supplemented with 5% fetal bovine serum (ScienCell Research Laboratories), 1% growth supplement (ScienCell Research Laboratories), and 1% penicillin/streptomycin solution (ScienCell Research Laboratories). After reaching confluence and waiting another 1–2 days, the preadipocytes were exposed to serum-free induction medium containing 0.5 mM 3-isobutyl-L-methylxanthine (Sigma-Aldrich), 1 μM dexamethasone (Sigma-Aldrich), 100 nM insulin (Sigma-Aldrich), 1 μM rosiglitazone (Sigma-Aldrich), and 1% penicillin/streptomycin solution for 4 days. Subsequently, the medium was replaced with the maintenance medium including DMEM/F-12 (Gibco), 100 nM insulin (Sigma-Aldrich), and 1% penicillin/streptomycin solution (Gibco) until the adipocytes were fully mature with and accumulated lipid droplet. The differentiated adipocytes were collected on days 4 and 8 for further detection.

To access FOXC2-AS1 expression level stimulated by forskolin (Fsk), differentiated adipocytes on days 4 and 8 were incubated in serum-free DMEM/F-12 medium for 6 h followed by stimulation with Fsk at a final concentration of 10 µM in DMEM/F-12 medium for additional 4–6 h to induce the thermogenic process in the adipocytes.

### Overexpression or Knockdown of lncRNA FOXC2-AS1 in Primary Human Preadipocytes

Overexpression of the full-length FOXC2-AS1 sequence was mediated by a lentivirus purchased from GenePharma Co. (Shanghai, China). The procedures were as follows. Once reaching 30%–40% confluence, the medium was exchanged with growth medium supplemented with FOXC2-AS1 overexpressing (FOXC2-AS1) or negative control (NC) lentivirus, respectively (virus titer, 10^6^ TU/mL) as well as Polybrene (5 μg/ml). The cells were recovered in PAM growth medium 16–18 h after transfection, and grown to confluence. FOXC2-AS1 expression was knocked down by the small interfering RNA (siRNA) method. Interfering sequences were specifically synthesized and purchased from Invitrogen Co. (Carlsbad, CA, USA) as follows: Si-1 CCGTTCAAGGTTTCCTTGCACCCTT;Si-2:CGGCTGCGTATTCGATTCTCAGCAA; Si-3: GGGCGTGCCACTTATTTCCAATAAA. A negative control sequence (moderate GC content) was also purchased from Invitrogen. When the preadipocytes reached 50%–60% confluence, they were transfected with siRNAs (Si-FOXC2-AS1) and the negative control (Si-NC) at 100 nM using Lipofectamine 3000 (Invitrogen) according to the manufacturer’s instructions. Upon reaching confluence and after 1–2 days, FOXC2-AS1 overexpressed- or knockdown-cells were induced to differentiate until they contained large lipid droplets and were harvested respectively on days 4 and 8 for the functional evaluation. The primary adipocyte sample size was three biologically independent samples from participants.

### RNA Extraction, Reverse Transcription, and Quantitative Real Time-Polymerase Chain Reaction (RT-PCR)

Total RNA from adipocytes was extracted with TRIzol reagent (Invitrogen). The RNeasy Mini Kit (Tiangen Biotech, Beijing, China) was used to extract and purify total RNA according to the manufacturer’s instructions. RNA (2,000 ng) from each sample was reverse transcribed into cDNA using the Scientific™ RevertAid Fist Strand cDNA Synthesis kit (Thermo Fisher Scientific). Quantitative RT-PCR was carried out using the QuantStudio™ 7 Flex Real-Time PCR System (Applied Biosystems, Foster, CA, USA) and the SYBR Green method (Life Technologies Corp., Gaithersburg, MD, USA) according to the manufacturer’s instructions. The results were presented by comparing the relative expression of genes to the internal reference PPIA by the 2^-ΔΔCT^ method. All primers are listed in **Supplemental Table S2**.

### Oil Red O Staining

Lipid accumulation was detected by Oil Red O staining on days 4 and 8 of differentiation program. The adipocytes were washed twice with PBS and then fixed for 30 min with 4% paraformaldehyde (Biosharp, Hefei, China). The cells were rinsed twice with PBS, stained with 0.2% (m/v) Oil Red O (Sigma-Aldrich) dissolved in isopropanol (Damao Chemical Reagent Factory, Tianjin, China) for 30 min at 37°C. The lipid droplets were evaluated by the Imager A2 fluorescence microscope (Carl Zeiss, Werk Gottingen, Germany) and representative figures were shown. To determine lipid content in the differentiated adipocytes on days 4 and 8, isopropanol (0.5ml/well of a 12-well plate) was added to the plates as reported previously (33, 34). After the extracted dye was removed *via* pipetting to a new 96-well plate (200 µl/well), and the optical density was detected spectrophotometrically at 510 nM with the Synergy H4 Hybird microplate reader (BioTek, Winooski, VT, USA).

### Protein Extraction and Western Blot

Total protein was isolated from cells using RIPA lysis buffer (Beyotime Biotechnology, Shanghai, China) containing 2% proteinase inhibitor cocktail (Roche, Basel, Switzerland). The protein samples were prepared according to the protein concentration determined by a BCA assay kit (Thermo Fisher Scientific). The candidate proteins were separated by 8%, 10%, or 12% sodium dodecyl sulfate-polyacrylamide gel electrophoresis based on molecular weight, transferred to a PVDF membrane (Millipore Corp., Billerica, MA, USA), and immunoblotted with specific primary antibodies as follows: anti-PRDM16 (Abcam, St. Louis, MO, USA; Cat. No. ab106410; diluted 1:1,000), anti-PGC1α (Abcam; Cat. No. ab54481; diluted 1:1,000), anti-UCP1 (Abcam; Cat. No. ab155117; diluted 1:1,000), anti-CEBPα (Abcam; Cat. No. ab40764; diluted 1:1,000), anti-PPARγ (Abcam; Cat. No. ab178860; diluted 1:1000), anti-ATGL (Cell Signaling Technology, Danvers, MA, USA; Cat. No. 2138S; diluted 1:1,000), anti-FOXC2 (Cell Signaling Technology; Cat. No. 12974S; diluted 1:1,000), anti-LC3 (Cell Signaling Technology; Cat. No. 12741S; diluted 1:1,000), and β-actin (Biosharp; Cat. No. BL005B; diluted 1:1,000). The second antibodies were: goat anti-mouse IgG HRP (Biosharp; Cat. No. BL001A; diluted 1:5,000) and goat anti-rabbit IgG HRP (Biosharp; Cat. No. BL003A; diluted 1:1,000). The gray values were scanned and calculated with ImageJ software (National Institutes of Health, Bethesda, MD, USA).

### Determination of Oxygen Consumption

The preadipocytes (5 × 10^5^ per well) were seeded in a specific 24-well plate (Agilent Technologies, Santa Clara, CA, USA) coated with 0.1% galectin (Sigma-Aldrich). The cells were induced into mature adipocytes as described above and the oxygen consumption rate (OCR) was determined on days 4 and 8 using the XF Cell Mito Stress Test Kit (Agilent Technologies) on an XF Extracellular Flux analyzer (Agilent Technologies) according to the manufacturer’s instructions. After detecting the basal OCR, the adipocytes were exposed to the following mitochondrial complex inhibitors: oligomycin (diluted to 1 μM, ATP synthase inhibitor), FCCP (diluted to 0.5 μM, uncoupling agent), and a rotenone/antimycin mixture (diluted to 0.5 μM, complex I and complex III inhibitor). Basal respiration, maximal respiration, ATP production, protein leak, and spare respiration were calculated according to the manufacturer’s instructions.

### RNA-Sequencing and Bioinformatics Analysis

Total RNA of the NC and FOXC2-AS1 groups from differentiated adipocytes (day 4) was extracted with TRIzol reagent. The Agilent 2100 Bioanalyzer (Agilent Technologies) was used to quantify and qualify total RNA in each sample. One μg of total RNA with a RIN > 7 was used in subsequent steps. Then, the RNAClean XP Kit (Beckman Coulter, Inc., Brea, CA, USA) and the RNase-Free DNase Set (Qiagen, GmBH, Hilden, Germany) were used to purify high quality total RNA. Next, the purified samples were subjected to RNA-seq analysis with the Agilent 2100 Bioanalyzer according to the Illumina User Guide. High quality clean data were filtered using Cutadapt (V1.9.1). The following data were processed using websites, such as NCBI (https://www.ncbi.nlm.nih.gov/), UCSC (http://genome.ucsc.edu/), ENSEMBL (http://asia.ensembl.org/index.html), and Hisat2 software (v2.0.1). An expression analysis was performed by HTSeq (v0.6.1), and the differentially expressed genes were analyzed using the DESeq2 Bioconductor package with |fold change| ≥ 1.5 and a *q*-value < 0.05. A Gene Ontology analysis was performed using GOSeq (v1.34.1) and the KEGG pathway analysis was performed at the website (http://en.wikipedia.org/wiki/KEGG).

### Inhibition of Autophagy Pathway by Treatment With 3-Methyladenine (3-MA)

To inhibit autophagy, the transfected adipocytes with siRNAs (Si-FOXC2-AS1) were treated by acute stimulation with the 3-MA (1 and 5 mM)(Sigma-Aldrich) or vehicle (DMSO) (Sigma-Aldrich) for additional 4 h. The treated cells were used for subsequent experiments at the indicated times.

### Statistical Analysis

The data analysis was performed using GraphPad Prism 7 (GraphPad Software, Inc., La Jolla, CA, USA) and expressed as mean ± standard deviation. Comparisons were made using the unpaired two-tailed *t*-test, and a *P-*value < 0.05 was considered significant.

## Results

### Characterization of lncRNA FOXC2-AS1 Involved in Human White Adipocyte Differentiation and the Browning Process

**Figure 1:**
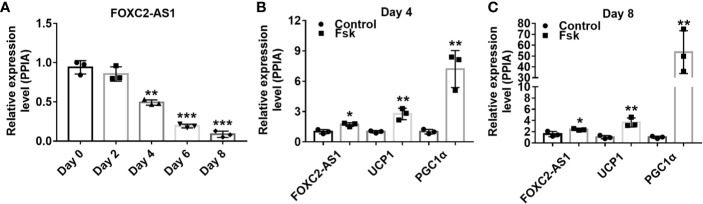
Characterization of lncRNA FOXC2-AS1 involved in white adipocyte differentiation and browning. **(A)** FOXC2-AS1 expression in human subcutaneous white adipocytes on day 0, day 2, day 4, day 6, and day 8 of differentiation program were detected by quantitative RT-PCR (n= 3 biological independent samples per time point). **(B, C)** FOXC2-AS1, UCP1, and PGC1a expression in differentiated white adipocytes stimulated with Fsk (10 μM) were evaluated by quantitative real time-polymerase chain reaction (RT-PCR) on day 4 and day 8 of differentiation program (n= 3 biological independent samples per group). PPIA served as internal controls for normalization. Values were presented as the mean ± SD. *P < 0.05, **P < 0.01, ***P < 0.001.

The FOXC2-AS1, also called RP11-463O9.5 (GenBank accession number, NR_027954), is located on chromosome 16 (16q24.1) and transcribed from the negative strand of forkhead box protein C2 (FOXC2) **(** **Figure S1A** **)**. FOXC2-AS1 is 319 bp in length and predicted to lack the ability to encode proteins according to the Coding Potential Assessment Tool (http://lilab.research.bcm.edu/cpat/index.php) **(** **Figure S1B** **)**. To study the effect of FOXC2-AS1 on adipogenesis, we analyzed its expression by quantitative RT-PCR during subcutaneous white adipocyte differentiation program. Compared to the undifferentiated preadipocytes (Day 0), the relative FOXC2-AS1 expression level decreased significantly with the adipogenic time **(** **Figure 1A** **)**. Fsk is a thermogenic agent that activates cAMP from ATP, thereby promoting fatty acid release from adipose tissue (35). To validate the expression pattern of FOXC2-AS1 during the white adipocyte browning process, we used Fsk (10 μM) to stimulate adipocytes respectively on days 4 and 8. The results showed that the expression levels of brown adipocyte-specific markers uncoupling protein 1 (UCP1) and peroxisome proliferator-activated receptor-γ coactlvator-1α (PGC1α) in the stimulated adipocytes increased significantly compared to those in unstimulated cells. Importantly, FOXC2-AS1 exhibited a significant increase of expression after stimulation with Fsk **(** **Figures 1B, C** **)**. These observations indicated that FOXC2-AS1 may play a role in regulating differentiation and browning processes in white adipocytes.

### LncRNA FOXC2-AS1 Has No Effect on Adipocyte Differentiation

**Figure 2:**
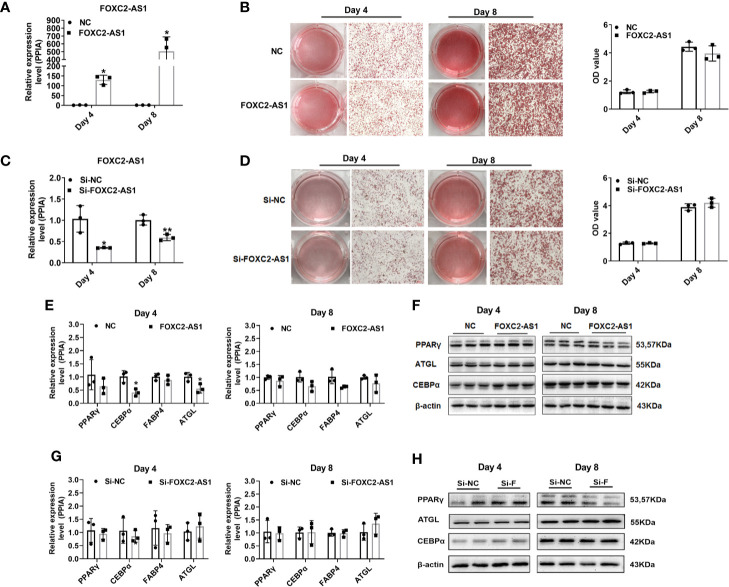
LncRNA FOXC2-AS1 has no effect on adipocyte differentiation. FOXC2-AS1 expression was overexpressed by lentivirus expressing FOXC2-AS1 or NC and knocked down using Si-FOXC2-AS1 or Si-NC in human subcutaneous white adipocytes (n= 3 biological independent samples per group). **(A, C)** Relative expression of FOXC2-AS1 on day 4 and day 8 of differentiation process was detected by quantitative quantitative real time-polymerase chain reaction (RT-PCR) after gain-or-loss of FOXC2-AS1. **(B, D)** Bright view of Oil Red O staining to evaluate lipid accumulation in white adipocytes on day 4 and day 8 of differentiation program after FOXC2-AS1 overexpression and knockdown. The representative views were shown. Oil Red O were also quantified spectrophotometrically and evaluated after FOXC2-AS1 overexpression and knockdown. **(E, F)** Common differentiation-related genes expression was detected by quantitative RT-PCR and western blot on day 4 and day 8 of differentiation program between FOXC2-AS1 overexpression and NC groups. **(G, H)** Common differentiation-related genes expression was detected by quantitative RT-PCR and western blot on day 4 and day 8 of differentiation program between FOXC2-AS1 knockdown and Si-NC groups. The representative immunoblotting graphs of western blot were shown. PPIA served as internal controls for normalization at mRNA level and β-actin served as internal controls for normalization at protein level. Values were presented as the mean ± SD. *P < 0.05, **P < 0.01.

To further reveal the possible role of FOXC2-AS1 in adipocyte differentiation, FOXC2-AS1 was overexpressed *via* lentivirus infection or knocked down *via* siRNA transfection respectively in human subcutaneous white adipocytes. Quantitative RT-PCR detection confirmed the FOXC2-AS1 expression level increased significantly in the overexpression group (FOXC2-AS1) compared to the NC group **(** **Figure 2A** **)**. Additionally, the interference efficiency of the Si-2 sequence on FOXC2-AS1 was the most significant among the three siRNAs’ (the efficiency of Si-1 and Si-3 were shown in **Figure S2**), which was reduced to 30% on day 4 and 40% on day 8 in the Si-FOXC2-AS1 group compared with the Si-NC group **(** **Figure 2C** **)**. The Oil Red O staining and the spectrophotometric results showed that FOXC2-AS1 overexpression or knockdown did not cause a detectable difference in lipid accumulation on days 4 or 8 during the differentiation program **(** **Figures 2B, D** **)**. Overexpression of FOXC2-AS1 on day 4 slightly affected the mRNA expression levels of common adipogenic-related markers, including CCAAT/enhancer binding protein alpha (C/EBPα) and adipose triacylglyceride lipase (ATGL) **(** **Figure 2E** **)**. However, C/EBPα and ATGL mRNA expression levels tended to be comparable between the two groups at the terminal stage of the differentiation program on day 8 **(** **Figure 2E** **)**. Knockdown of FOXC2-AS1 consistently did not alter the mRNA levels of any of the adipogenic-related genes **(** **Figure 2G** **)**. Furthermore, the PPARγ, C/EBPα, and ATGL protein levels did not change significantly after FOXC2-AS1 overexpression **(** **Figure 2F** **)** or knockdown **(** **Figure 2H** **)**. Additionally, phosphorylation levels of ATGL and AMPK were detected after FOXC2-AS1 overexpression or knockdown to verify their lipolytic activity and increased p-ATGL/ATGL and p-AMPK/AMPK ratios were only observed in adipocytes after FOXC2-AS1 overexpression on day 4 **(** **Figure S3** **)**. Overall, our results demonstrate that FOXC2-AS1 is not required for adipogenic processing but promotes lipolytic activity of human subcutaneous white adipocytes.

### LncRNA FOXC2-AS1 Regulates the Browning Process in Human Subcutaneous White Adipocytes

**Figure 3:**
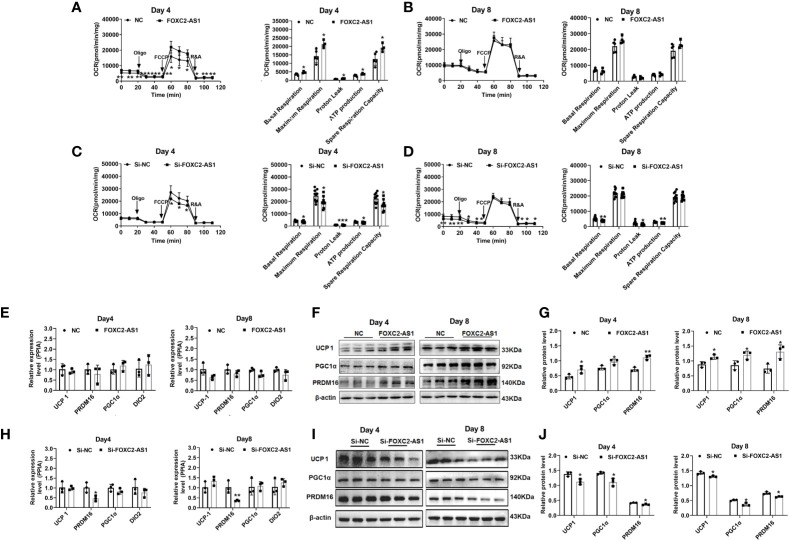
LncRNA FOXC2-AS1 regulates browning process of white adipocytes. Oxygen consumption determination was used to detect mitochondria function on day 4 and day 8 of adipogenic differentiation program. The differentiated white adipocytes from FOXC2-AS1 overexpression and NC groups **(A, B)** groups as well as Si-FOXC2-AS1 and Si-NC groups **(C, D)** were treated with respiratory inhibitors as following Oligo, FCCP, and R&A to dissect the various parts of the respiration program. Quantitation of the oxygen consumption rates (OCRs) including basal respiration, ATP production, maximal respiration, proton leak, and spare respiration capacity. n=5 or 9 technical replicates. **(E–J):** Brown adipocyte-specific markers were detected by quantitative RT-PCR and western blot after FOXC2-AS1 overexpression and knockdown. The representative immunoblotting graphs of western blot were shown. PPIA served as internal controls for normalization at mRNA level and β-actin served as internal controls for normalization at protein level. n=3 biological independent samples. Values were presented as the mean ± SD. *P < 0.05, **P < 0.01, ***P < 0.001.

To determine whether FOXC2-AS1 is involved in regulating of human white adipocyte browning, we detected the browning phenotype after FOXC2-AS1 overexpression or knockdown. FOXC2-AS1-overexpressing cells had a significantly higher thermogenic capacity compared to the NC group, as measured by the OCR on day 4 of the differentiation program **(** **Figure 3A** **)**. Cellular metabolic parameters, including basal respiration, maximal respiratory respiration, ATP production, protein leak, and spare respiration increased after FOXC2-AS1 overexpression **(** **Figure 3A** **).** However, at the terminal stage of the differentiation program on day 8, OCR rates and the above parameters became equal between the NC and FOXC2-AS1 overexpressing groups **(** **Figure 3B** **)**. When FOXC2-AS1 was knocked down, a downregulated trend in cellular metabolic parameters, including basal respiration, protein leak, ATP production, and spare respiration was observed on days 4 and 8 **(** **Figures 3C, D** **)**. Accordingly, the protein levels of brown adipocyte-specific markers, such as UCP1, PGC1α, and positive regulatory domain containing 16 (PRDM16), were upregulated **(** **Figures 3F, G** **)**, while these mRNA expression levels did not change in human subcutaneous white adipocytes after FOXC2-AS1 overexpression **(** **Figure 3E** **)**. FOXC2-AS1 knockdown presented a dramatic decrease in the PRDM16 mRNA expression level **(** **Figure 3H** **)** and resulted in the decreased protein levels in UCP1, PGC1α, and PRDM16 protein expression levels **(** **Figures 3I, J** **)**. These observations indicate that FOXC2-AS1 may promote the white-to-brown adipocyte conversion by affecting thermogenic capacity and mitochondrial content.

### LncRNA FOXC2-AS1 Regulates White Adipocyte Browning Through the Autophagy Signaling Pathway

**Figure 4:**
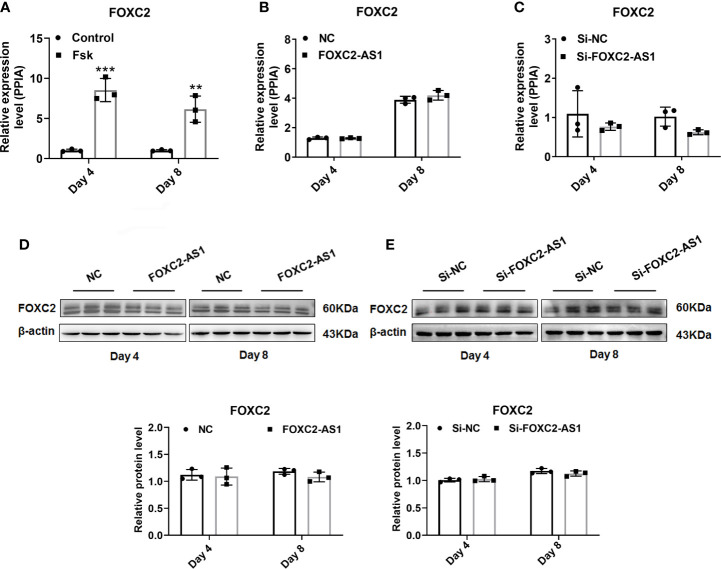
FOXC2 does not participate in the functional influence by lncRNA FOXC2-AS1. **(A)** Expression levels of FOXC2 mRNA on day 4 and day 8 of differentiation program were detected by quantitative real time-polymerase chain reaction (RT-PCR) after treatment with Fsk, and the cells with dilute solution served as Control group (n= 3 biological independent samples per group). **(B, C)** FOXC2 mRNA expression levels were detected after overexpression or knockdown of FOXC2-AS1 on day 4 and day 8 of differentiation program. **(D, E)** FOXC2 protein levels were evaluated after overexpression or knockdown of FOXC2-AS1 on day 4 and day 8 of differentiation program. PPIA served as internal controls for normalization at mRNA level and β-actin served as internal controls for normalization at protein level. Data are mean ± SD. **P < 0.01; ***P < 0.001.

**Figure 5:**
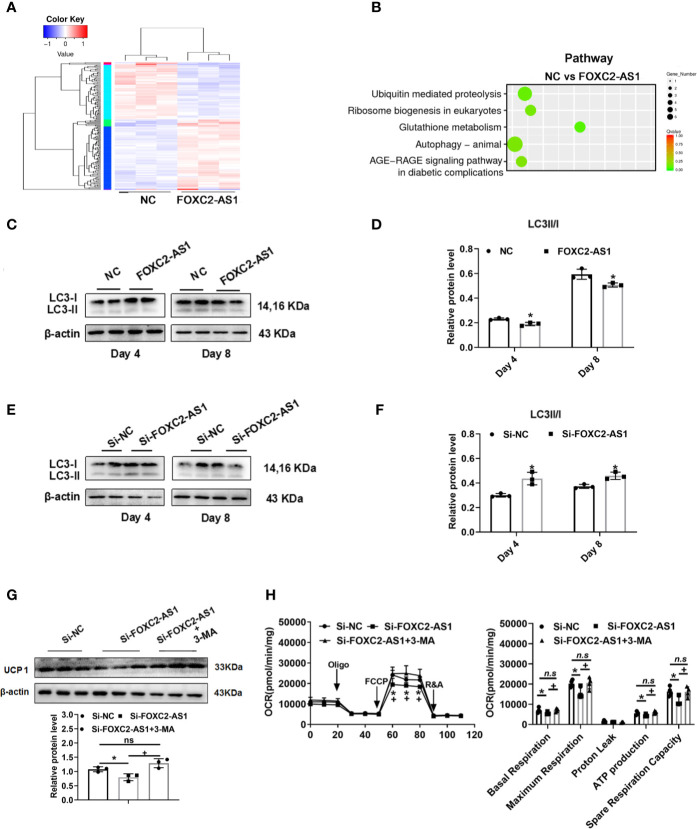
LncRNA FOXC2-AS1 may regulate white adipocyte browning through the autophagy signaling pathway. **(A)** Heatmap of differentially expressed genes between FOXC2-AS1 overexpression and NC groups in white adipocytes (n= 3 biological independent samples). **(B)** Pathway analysis of genes that were differentially expressed after FOXC2-AS1 overexpression in white adipocytes. **(C, E)** Protein levels of autophagy-related marker LC3 were detected by western blot after overexpression or knockdown of FOXC2-AS1 on day 4 and day 8 of differentiation. **(D, F)** Gray analysis of autophagy-related marker LC3 proteins and calculation of the ratio of LC3II to LC3I after overexpression or knockdown of FOXC2-AS1 on day 4 and day 8 of differentiation program. **(G)** Protein levels and gray analysis of UCP1 were evaluated in the differentiated adipocytes on day 4 from Si-FOXC2-AS1 groups treated with or without autophagy inhibitor 3-MA (5 mM) for additional 4 h. **(H)** OCRs of the differentiated adipocytes from Si-NC, Si-FOXC2-AS1 and Si-FOXC2-AS1 groups treated with 3-MA (5 mM) for additional 4h were evaluated. Quantitation of OCRs including basal respiration, ATP production, maximal respiration, proton leak, and spare respiration capacity. n=4 technical replicates per group. The representative immunoblotting graphs of western blot were shown of 3 biological independent samples. β-actin served as internal control. Values were presented as the mean ± SD. *P < 0.05 compared between the Si-NC and Si-FOXC2-AS1 groups. +*P* < 0.05 compared between Si-FOXC2-AS1 and Si-FOXC2-AS1 groups treated with 3-MA groups. “n.s.” means no significance.

Several studies have reported that antisense lncRNAs can affect regulatory function by affecting their neighboring protein-encoding gene expression (36). Thus, we first examined the association between FOXC2-AS1 and its neighboring gene FOXC2. Quantitative RT-PCR results indicated that the FOXC2 expression level in response to Fsk was significantly upregulated in stimulated cells **(** **Figure 4A** **)**, showing a consistent FOXC2-AS1expression trend **(** **Figures 1B, C** **)**. Next, we tried to determine whether FOXC2-AS1 affected FOXC2 expression. However, overexpression or knockdown of FOXC2-AS1 did not alter FOXC2 mRNA **(** **Figures 4B, C** **)** or protein levels **(** **Figures 4D, E** **)**. We employed RNA-sequencing to search for a possible regulatory mechanism and reveal the mechanistic insight underlying the white-to-brown adipocyte conversion by FOXC2-AS1. Considering the browning phenotype on day 4 after FOXC2-AS1 overexpression, the differentially expressed genes (DEGs) between FOXC2-AS1-overexpressed and NC white adipocytes were profiled. A total of 147 DEGs were detected (|fold change| > 1.5, *p* < 0.05) as shown in **Figure 5A**. Sixty-six genes were upregulated and 81 genes were downregulated in the FOXC2-AS1 group compared to the NC group **(** **Supplemental Table S3** **)**. By querying these DEGs in the KEGG pathway analysis, these genes were mainly enriched in ubiquitin-mediated proteolysis, ribosome biogenesis, glutathione metabolism, the autophagy signaling pathway, and the AGE-RAGE signaling pathway **(** **Figure 5B** **and** **Supplemental Table S4** **)**. Notably, mounting evidence has demonstrated that inhibiting the autophagy signaling pathway promotes the conversion of white to brown adipocytes (37–39). To validate whether autophagy is involved in the browning program of white adipocytes mediated by FOXC2-AS1, we evaluated the autophagy-specific protein microtubule associated protein 1 light chain 3 (MAP1LC3; LC3) after FOXC2-AS1 overexpression or knockdown by western blot. As results, the LC3 II/LC3 I ratio decreased significantly after FOXC2-AS1 overexpression **(** **Figures 5C, D** **)** but increased after FOXC2-AS1 knockdown **(** **Figures 5E, F** **)** on days 4 and 8 of adipogenic differentiation. To explore if FOXC2-AS1-regulated white adipocyte browning was mediated by affecting the autophagy signaling pathway, adipocytes were acutely stimulated with the autophagy inhibitor 3-MA (DMSO, 1 and 5 mM) when FOXC2-AS1 expression was inhibited. As a result, 3-MA (5 mM) significantly inhibited autophagy **(** **Figure S4A** **)**. Acute stimulation with 3-MA did not affect lipid accumulation **(** **Figure S4B** **)**. Additionally, 3-MA restored the reduced UCP1 protein level and thermogenic capacity caused by inhibiting FOXC2-AS1 **(** **Figures 5G, H** **)**. The inhibition of autophagy in adipocytes attenuated the inhibitory effects of FOXC2-AS1 on adipocyte browning, indicating that FOXC2-AS1 may mediate the adipocyte thermogenic program by regulating the adipocyte autophagy level.

## Discussion

Accumulating evidence has demonstrated that lncRNAs are involved in regulation of adipocyte differentiation and functions (40). The functional influence of lncRNAs in the aspect of adipocyte differentiation is relatively definite. As reported, lncRNAs, such as HOTAIR (41), ADINR (42), MIR31HG (25), ASMER-1, ASMER-2 (43), HOXA11-AS1 (44), and linc-ADAL promote the differentiation of white adipocytes, while MEG3 (45) have been demonstrated to have an inhibitory effect on white adipocyte differentiation. However, few studies have focused on the potential roles of lncRNAs involved in the browning process of white adipocytes.

In combination with our results, FOXC2-AS1 expression continued to decline during the differentiation program, suggesting a potential role of FOXC2-AS1 in regulating adipocyte differentiation program as shown in **Figure 1A**. Furthermore, FOXC2-AS1 expression increased significantly in human white adipocytes stimulated with Fsk as presented in **Figures 1B, C**. Fsk is a classical inductor of browning of adipocytes, which triggers cAMP production. Much progress has been made in elucidating the cAMP-regulated genes, including interferon regulatory factor 4 (46), bone morphogenic protein 7 (47), zinc finger protein 516 (48), and zinc finger protein 638 (49), which promotes the browning characteristics of white adipose with a more oxidative metabolic phenotype. In addition to encoding genes, adipose tissues- or adipocytes- derived lncRNA BATE10 (28) and Gm13133 (30) have also been demonstrated to be regulated by cAMP, showing a similar expression pattern with FOXC2-AS1. cAMP immediately activates protein kinase A and phosphorylation of cAMP response element binding protein (CREB) (50, 51). The activated CREB and its family members recruiting some coactivators and stimulates downstream thermogenic gene expression by directly binding at the cAMP response element (CRE) site of the gene promoter (50, 51). Thus, we speculate that FOXC2-AS1 may be induced by this mechanism in white adipocytes. Further analysis of CRE enrichment sites of the FOXC2-AS1 promoter region and validation of the regulatory mechanism by the luciferase reporter gene assay need to be conducted to reveal the possible molecular mechanism of FOXC2-AS1 underlying its induction by cAMP.

The change in FOXC2-AS1 during differentiation and the thermogenic program persuaded us to confirm its possible role in white adipocytes. From our results, gain-of-FOXC2-AS1 was sufficient to promote the browning program of white adipocytes in **Figures 3A, F, G**, but not in lipid accumulation, as shown in **Figure 2B**. FOXC2-AS1-overexpressing cells had equal thermogenic capacity but an upregulated UCP1 protein expression level compared to the NC group on day 8, which was an inconsistent finding **(** **Figure 3B** **)**. This inconsistent finding may be explained that the increased levels of browning proteins were insufficient for the changed phenotype. Stimulation with the thermogenic agent Fsk may be a more useful approach to explore the role of FOXC2-AS1 in the white-to-brown conversion in future research. Accordingly, knockdown of FOXC2-AS1 also did not affect lipid accumulation or adipogenic marker genes expression as presented in **Figures 2D, G**, but it did significantly inhibit the browning process as presented in **Figures 3C, D, H–J**. Given the prominent role of ATGL (52) and AMPK (53) in adipocyte lipolysis, which provides free fatty acids as energy substrate, increased ATGL and AMPK phosphorylation levels were observed, indicating upregulated lipolytic activity due to FOXC2-AS1 overexpression **(** **Figure S3A** **)**. However, this effect was not observed after inhibiting FOXC2-AS1, further demonstrating that this lncRNA is not required to regulate lipolytic activity in adipocytes.

Overall, our results demonstrate that FOXC2-AS1 is not required for adipogenic processing. Thus, our observations suggest that FOXC2-AS1 is not required for adipocyte differentiation and no additional coactivators are needed to achieve its functional effect. More importantly, some authors have reported the importance and the specific regulation of protein factors merely in the thermogenic program or the browning process. For example, KDM1 lysine (K)-specific demethylase 6B mediated Ucp1 and Cidea expression but not for adipogenesis, and facilitates browning of white adipocytes (54). Another protein called histone deacetylase 3 has also been reported neither triggered nor required for brown adipocytes differentiation, but involved in modulating expression of Ucp1 and mitochondrial OXPHOS and TCA cycle genes (55). In addition to these encoding genes, the functional effect of lncRNA BATE1 and BATE10 was not associated with a significant effect on lipid accumulation but with a mild effect on common adipogenic gene expression (28, 56). Interestingly, a study of mRNAs screened after BATE10 knockdown revealed a remarkably enriched respiratory electron transport pathway, which is considered a hallmark of BAT function (56). This observation shows that the FOXC2-AS1 regulatory mechanism may be associated with the signaling pathways or key regulators involved in the thermogenic program or browning of white adipocytes.

The regulatory mechanisms of lncRNAs are complex and are closely related to their sequence, position, and structural characteristics (40). Recent studies have validated the regulatory relationship between antisense lncRNAs and their neighboring genes (36). FOXC2-AS1 is an antisense lncRNA transcribed from the negative strand of the FOXC2 gene locus. The genomic proximity of FOXC2-AS1 and FOXC2 along with the functional effect of FOXC2 on the induction of adipocytes mitochondriogenesis (57) and activation of the signal transducer and activator of transcription 3-PRDM16 signal (58) inspired us to explore the regulatory relationship between them. Although FOXC2 presented an increased expression pattern after Fsk stimulation, mRNA and protein levels did not change significantly by gain-or loss-of-FOXC2-AS1, as shown in **Figures 4B–E**. When we determined the downstream regulatory mechanism by gain-of-FOXC2-AS1 at the genome-wide level, the inhibited autophagy pathway attracted our attention, as shown in **Figure 5**. The autophagy signaling pathway controls adipocyte differentiation and balances the transition between white and brown adipocytes. Inhibiting autophagy by knockout of autophagy-related genes not only suppresses lipid accumulation, but also promotes white adipocyte browning in WAT (39, 59). Additionally, deficiencies in autophagy also led to higher mitochondrial content and brown adipocytes-specific proteins levels to maintain thermogenic adipocytes identity by suppressing clearance of mitochondria (37, 60). In contrast, autophagy-related genes are activated under an obese status (61) and activation of autophagy by pharmacological stimulation, such as dexamethasone, could result in the opposite phenotype (62). LC3 is considered an autophagy-specific marker for evaluating autophagy. During autophagy, autophagosomes engulf cytoplasmic proteins and organelles, and combine cytosolic form LC3 (LC3-I) with phosphatidyl ethanolamine to form LC3-phosphatidyl ethanolamine conjugation (LC3-II), which is collected on autophagosome membrane (63). Overexpressing FOXC2-AS1 decreased the LC3 II/LC3 I ratio (**Figures 5C, D** **)**, while FOXC2-AS1 knockdown increased this ratio (**Figures 5E, F** **)**. The reduction of UCP1 was suppressed and thermogenic capacity was inhibited when the autophagy inhibitor 3-MA was administered while inhibiting FOXC2-AS1 **(** **Figures 5G, H** **)**. This finding further elucidates that FOXC2-AS1 regulates adipocytes browning by affecting autophagy. 3-MA has also been used to explore the relationship between novel adipokine CTRP5 and adipocytes browning (64). Gain-or loss-of FOXC2-AS did not regulate adipocyte differentiation; however, one of the functions of autophagy is to control normal adipogenesis. Considering this inconsistency, autophagy may be partly involved in the FOXC2-AS1 regulatory mechanism underlying the browning process, but additional factors need to be identified that are associated with the browning effect.

In summary, our present study highlights the role of FOXC2-AS1 in human subcutaneous white adipocytes. FOXC2-AS1 may promote the white-to-brown adipocyte transition partly by inhibiting the autophagy signaling pathway. This study identified a novel lncRNA regulatory network to control the white-to-brown adipocyte transition; thus, providing a therapeutic opportunity for preventing and treating obesity and its related metabolic disorders.

## Data Availability Statement

The original contributions presented in the study are included in the article/**Supplementary Materials**. Further inquiries can be directed to the corresponding authors.

## Ethics Statement

This study was approved by the Human Research Ethics Committee of Nanjing Maternity and Child Health Care Hospital (permit number [2020]KY-027). Signed informed consent for the tissue was obtained.

## Author Contributions

YW and SH performed experiments and interpreted results of experiments. XWC and YC prepared the figures. JW analyzed the data. XC and CJ participated in the discussion. LY and LP conceived and designed experiments, provided main funding to regents, and approved the final version of the manuscript. All authors contributed to the article and approved the submitted version.

## Funding

This study was supported by the National Natural Science Foundation of China (Grant No. 81600687, 81770866, 81800772), the Jiangsu Province Natural Science Foundation (Grant No. BK20191126 and BK20180146), the Jiangsu Provincial Medical Innovation Team Program (Grant No. CXTDA 2017001), the Nanjing Medical Science and Technique Development Foundation (Grant No. QRX17160), the Science and Technology Development Foundation Item of Nanjing Medical University (Grant No.2017NJMUZD074), and the “six talent peak” High-level Talents Training Project of Jiangsu Province (Grant No. C-YY-081).

## Conflict of Interest

The authors declare that the research was conducted in the absence of any commercial or financial relationships that could be construed as a potential conflict of interest.

## Abbreviations

ATGL, adipose triacylglyceride Lipase; ADINR, CEBPA divergent transcript; ASMER-1, adipocyte specific metabolic related lncRNA 1; ASMER-2, adipocyte specific metabolic related lncRNA 2; BAT, brown adipose tissue; BATE10, brown adipose tissue enriched long non-coding RNA 10; Blnc1, brown fat lncRNA 1; BMI, Body Mass Index; C/EBPα, CCAAT/enhancer binding protein Alpha; C/EBPβ, CCAAT/enhancer binding protein Beta; cAMP, cyclic adenosine monophosphate; CRE, cAMP response element; CREB, cAMP response element binding protein; DEGs, differentially expressed genes; FOXC2, forkhead box protein C2; FOXC2-AS1, forkhead Box C2 antisense RNA 1; Fsk, forskolin; H19, H19 imprinted maternally expressed transcript; HOTAIR, hox transcript antisense RNA; HOXA11-AS1, hoxa11 antisense RNA; LC3, light chain 3; LC3-I, cytosolic form LC3; LC3-II, autophagosome form LC3; linc-ADAL, lincRNA adipogenesis and lipogenesis associated; lncRNAs, long non-coding RNAs; 3-MA, 3-methyladenine; MAP1LC3, microtubule associated protein 1 light chain 3; MEG3, maternally expressed 3; MIR31HG, MIR31 host gene; NC, negative control; OCR, oxygen consumption rate; OD, optical density; Oligo, oligomycin; PGC1α, peroxlsome proliferator-activated receptor-γ coactlvator-1α; PRDM16, positive regulatory domain containing 16; R&A, rotenone/antimycin mixture; RT-PCR, quantitative real time-polymerase chain reaction; SD, standard deviation; siRNA, small interfering RNA; UCP1, uncoupling protein 1; WAT, white adipose tissue.
