# Supplementary figures and images for: The Effect of FOXC2-AS1 on White Adipocyte Browning and the Possible Regulatory Mechanism
Source: Front Endocrinol (Lausanne). 2020 Oct 29;11:565483. doi: 10.3389/fendo.2020.565483 (PMC7658007; doi:10.3389/fendo.2020.565483)

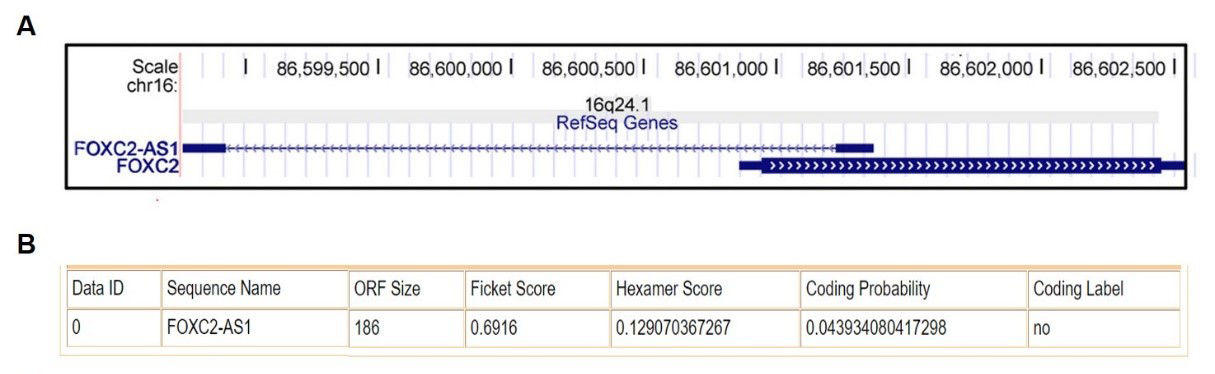

Supplement: Supplementary file 1 [file Image_1.tif]

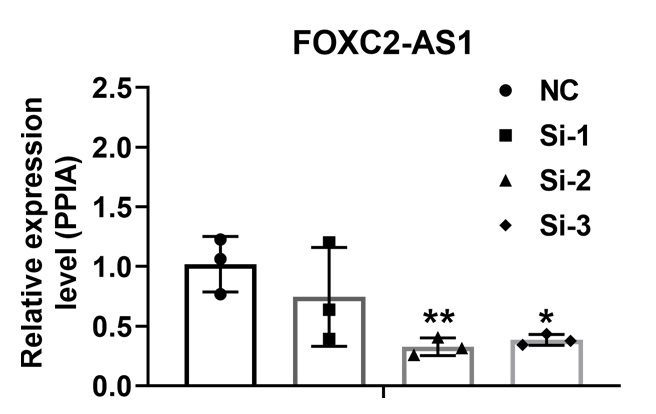

Supplement: Supplementary file 2 [file Image_2.tif]

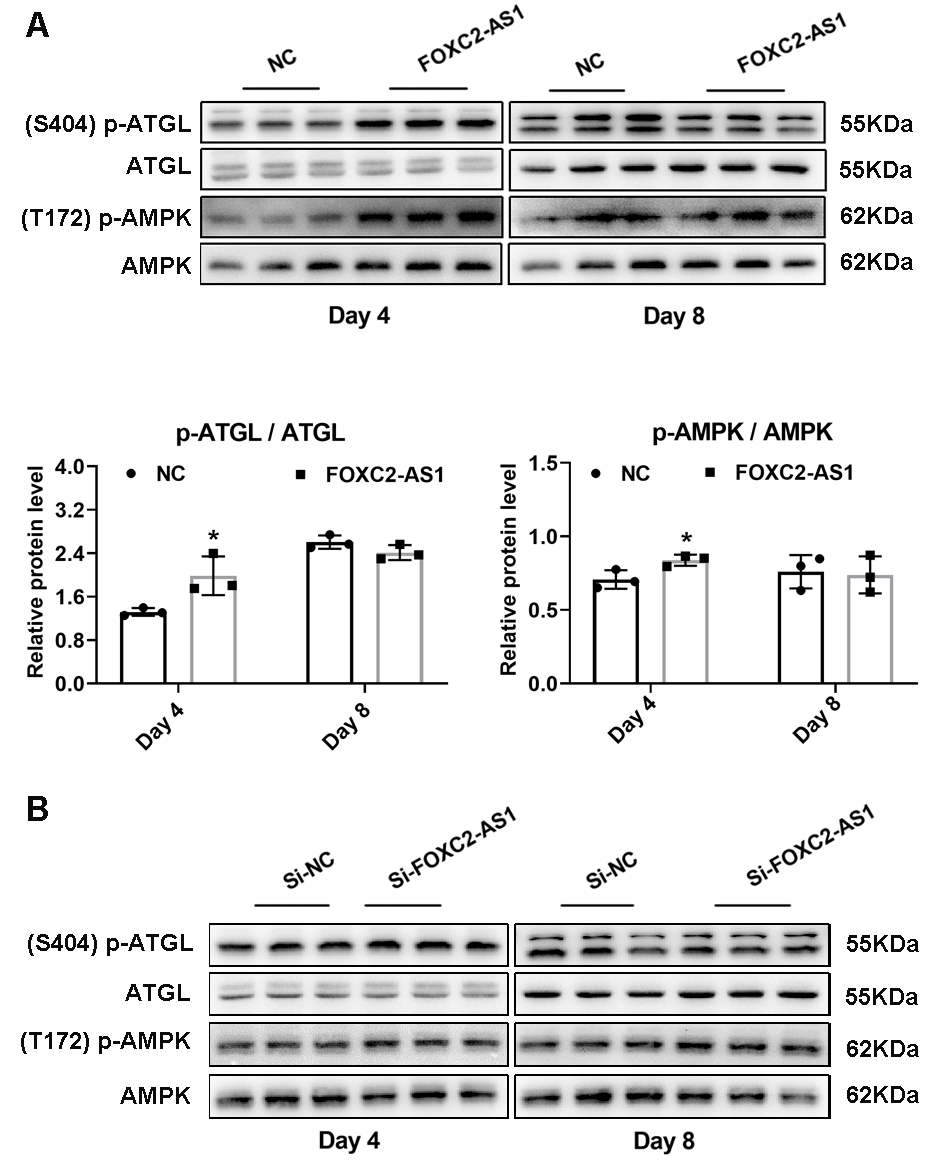

Supplement: Supplementary file 3 [file Image_3.tif]

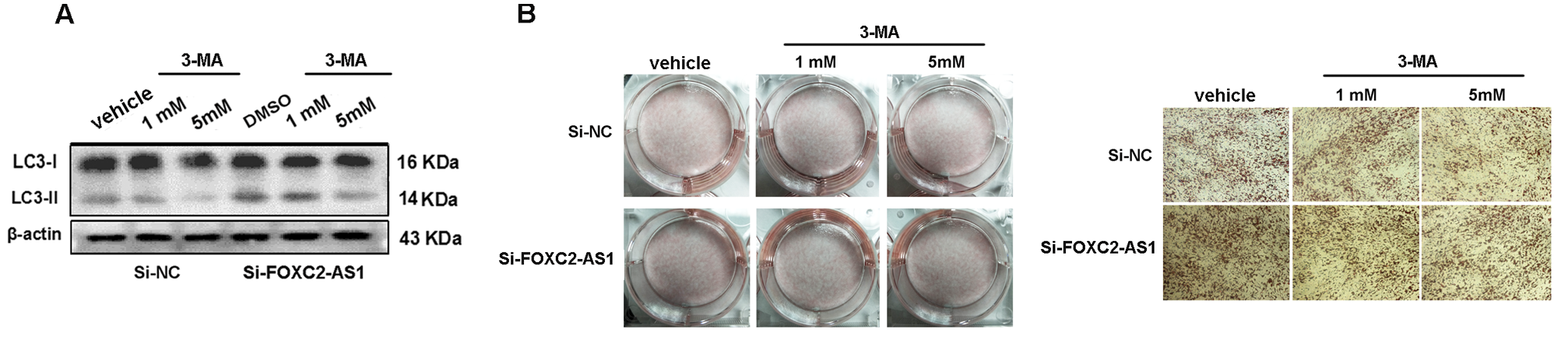

Supplement: Supplementary file 4 [file Image_4.tif]
