# Supplementary material for: The Effect of FOXC2-AS1 on White Adipocyte Browning and the Possible Regulatory Mechanism
Source: Front Endocrinol (Lausanne). 2020 Oct 29;11:565483. doi: 10.3389/fendo.2020.565483 (PMC7658007; doi:10.3389/fendo.2020.565483)
Supplement: Supplementary file 5 [file Table_1.docx]

Supplemental Table 1. The information of candidates used for the subcutaneous fat isolation

| Age | Gender | Weight(kg) | Height(cm) | BMI |
| --- | --- | --- | --- | --- |
| 34 | Female | 66.2 | 165 | 24.31589 |
| 36 | Female | 74.6 | 171 | 25.51212 |
| 29 | Female | 69.1 | 158 | 27.67986 |
